# Describe Your Feelings: Body Illusion Related to Alexithymia in Adolescence

**DOI:** 10.3389/fpsyg.2016.01690

**Published:** 2016-10-28

**Authors:** Eleana Georgiou, Sandra Mai, Olga Pollatos

**Affiliations:** Clinical and Health Psychology, Institute of Psychology and Education, Ulm UniversityUlm, Germany

**Keywords:** body illusion, rubber hand, alexithymia, emotions, adolescents

## Abstract

**Objective:** Having access to bodily signals is known to be crucial for differentiating the self from others and coping with negative feelings. The interplay between bodily and emotional processes develops in adolescence, where vulnerability is high, as negative affect states often occur, that could hamper the integration of bodily input into the self. Aim of the present study in healthy adolescents was to examine, whether a disturbed emotional awareness, described by the alexithymic construct, could trigger a higher malleability in the sense of body-ownership.

**Methods:** Fifty-four healthy adolescents aged between 12 to 17 years participated in this study. The Strength and Difficulties Questionnaire (SDQ) and the Screening psychischer Störungen im Jugendalter were used to assess emotional distress and conduct problems. Alexithymia was assessed by the TAS-20. The rubber hand illusion was implemented for examining the malleability of body-ownership.

**Results:** A higher body illusion was found to be connected with “difficulties in describing feelings”. Moreover, a higher degree of self-reported conduct and emotional problems as assessed by the SDQ were associated with a more pronounced body illusion. Further findings revealed an association between emotional distress and the emotional alexithymia subscales “difficulties in identifying feelings” and “difficulties in describing feelings”.

**Conclusion:** Our findings emphasize a close link between the sense of body-ownership and emotional awareness as assessed by emotional facets of the alexithymic trait. We suggest that in adolescents with higher malleability of body-ownership, a vicious circle might occur where affect and integration of different proprioceptive signals regarding the body become more entangled.

## Introduction

Adolescence is the time where major physical and psychological developmental changes are taking place. It is often known as a transitional stage, where the sense of identity or the establishment of a social role is being formatted/developed and the emotional autonomy or cognitive independence plays a crucial role (Maldonado et al., 2013). Body image and self-esteem are often pertinent in this stage and mass media are influential (Patton et al., 2014). Accordingly, eating disorders or other mental disorders are usually being firstly expressed during adolescence and early adulthood (Preti et al., 2009; Benjet et al., 2015). Therefore, the exploration of these changes in this transitional developmental phase is important.

Recent studies investigated the sense of body-ownership (perception of body as mine) using the rubber hand illusion (RHI) experiment, mostly in adults; in this paradigm participants estimate if a rubber hand, which had been synchronously stimulated with one’s own unseen hand, feels like their own hand (Tsakiris et al., 2011; Suzuki et al., 2013). Multisensory information, arising from vision, touch and proprioception, is the key for forming the bodily self, where visual-tactile processes are central for the body-ownership and mature during early childhood (Cowie et al., 2013). On the contrary, visual-proprioceptive processes, which are determinable for the visual-tactile integration in adults develop later in childhood (Cowie et al., 2013). Nonetheless, forming the bodily self, having access to bodily signals and differentiating the self from the others is crucial in the developmental processes in order to form more complex social behaviors, such as: social referencing, imitation, empathy, dealing with social exclusion or ostracism (Gallese, 2003; Chaminade et al., 2005; Cascio et al., 2012; Pollatos et al., 2015).

A previous study regarding the RHI in adults and children revealed a dissociation between the sense of body-ownership/ the experience of body as “mine” (Tsakiris, 2010) and the visual perception of hand position in children (Cowie et al., 2013). Moreover, proprioceptive drift responses were influenced by age, suggesting that children 4 to 9 years old indicated greater proprioceptive drifts than adults across synchronous and asynchronous conditions (Cowie et al., 2013). Likewise, another study investigated the role of body-ownership among children with autism spectrum disorders, revealing that autistic children demonstrated a delayed perception of the illusion in comparison to children with normal development. The authors concluded that the integration of visual and tactile information for the formation of body perception is a multidimensional process characterized by distinct differences among healthy and clinical populations (Cascio et al., 2012).

The construct of alexithymia is of great importance in this context: Alexithymia (“no words for feelings”), first described by Sifneos (1976), refers to a personality trait characterized by an inability to describe, express and identify one’s and others’ feelings as well as an externally oriented thinking style (Sifneos, 1976; Herbert and Pollatos, 2012). Alexithymia is related to a wide range of mental disorders (Taylor et al., 1999) including eating disorders (de Zwaan et al., 1996; Taylor et al., 1999; Corcos et al., 2000; Berthoz et al., 2007; Pollatos et al., 2008), depression (Taylor and Bagby, 2004; Foran and O’Leary, 2013; Panayiotou et al., 2015), anxiety disorders (Turk et al., 2005), substance use (Lyvers et al., 2013) and a variety of somatic illnesses such as diabetes mellitus (Chatzi et al., 2009). Self-report measures like the Toronto Alexithymia Scale (Bagby et al., 1994), the most widely used and well-validated assessment tool (Bagby et al., 1994; Parker et al., 2003; Mattila et al., 2007), assess alexithymia and its three main facets; namely difficulties in identifying feelings (TAS 1, DIF), difficulties in describing feelings (TAS2, DDF), and externally oriented thinking or a preoccupation with the details of external events (TAS3, EOT). There is empirical evidence that facets of alexithymia interact with the prevalence of dissociation (Tolmunen et al., 2010) and the risk for developing a psychosis (van’t Wout et al., 2007; van der Velde et al., 2015), suggesting that core elements of alexithymia may support negative affect states that hamper the integration of bodily input into the self and might also affect the sense of body-ownership.

This assumption is in accordance with data of Herbert and colleagues (Herbert et al., 2011) who reported less accurate ability to perceive bodily signals in alexithymia. Tsakiris et al. (2011) could show that participants with less interoceptive accuracy have a higher malleability of body-ownership processes as measured by the RHI paradigm. In spite of these data, empirical research examining a possible relationship between alexithymic trait and body-ownership is to our knowledge sparse. There is one recent study by Grynberg and Pollatos (2015) demonstrating a higher malleability of body-ownership in adults with higher scores of alexithymia. The sense of body-ownership in relation to alexithymia was to our knowledge not examined in adolescents so far, though the prevalence of alexithymia in younger adolescents is relatively high (Säkkinen et al., 2007).

All the above could suggest that the sense of body-ownership and alexithymia could be connected and this could have implications for some mental and somatic symptoms suffered by adolescents. Thus, scope of this study was to investigate the interrelation between alexithymia and its constructs with body-ownership. Based on current literature, we hypothesized that higher degrees of alexithymia would be connected to a more pronounced malleability of body-ownership as measured by a stronger body-illusion in healthy adolescents.

## Materials and Methods

### Participants

In total, our sample consisted of 54 healthy participants fulfilling all required criteria, such as absence of a diagnosed psychological disorder, or other medical condition (e.g.: cardiovascular disease, bronchial asthma, diabetes etc.). More specific 28 girls and 26 boys, aged between 12 to 17, took part in the study, with a mean age of 14.0 years (*SD* = 1.55) and a mean BMI of 19.7 (*SD* = 2.8). Flyers were distributed in sports and chess clubs; advertisements were placed in local newspapers. All experiments were conducted in accordance to the Declaration of Helsinki and were approved by the Ethics Committee of Ulm University.

### Experimental Procedure

The participants and their parents gave their written informed consent and filled in online several questionnaires, which are described below. The study took place in the laboratory of the Clinical and Health Psychology Department of Ulm University. The experimental procedure, including the RHI and other tasks not being reported here, lasted about 90 min and all participants received a cinema voucher for their participation.

### Questionnaires

#### Strength and Difficulties Questionnaire (SDQ)

The Strength and Difficulties Questionnaire (SDQ) is a brief behavioral screening instrument for children and adolescents, which has already been translated into 40 languages. It is divided into five subscales: conduct problems, hyperactivity-inattention, emotional problems, peer problems, and prosocial behavior. In this study we used the German self-report version and more specifically the total score, in which the first four subscales are summed up. The German SDQ was found to correlate highly with the Child Behavior Checklist (CBCL) (Klasen et al., 2000). The psychometric properties of the German sum score SDQ demonstrated an internal consistency of α = 0.82 (Rothenberger et al., 2008).

#### Screening Psychischer Störungen im Jugendalter (SPS-J)

The SPS-J is a screening questionnaire for psychological disorders in adolescence, which is the German translation of the Reynolds Adolescent Adjustment Screening Inventory (RAASI) and is suitable for assessing emotional and conduct problems. It is comprised of the following subscales: antisocial behavior, anger control problems, emotional distress and negative self. For this study we used only the scale emotional distress in order to evaluate the degree of depression or sadness and anxiety. Internal consistency measured by Cronbach α for this test lies between.75 to.84 and repeatability after six weeks was found to be satisfactory (*r*_tt_ = 0.55 to 0.73) (Hampel and Petermann, 2006).

#### Alexithymia

Alexithymia was measured by the Toronto Alexithymia Scale (TAS-20). The TAS-20 is the most commonly used self-report questionnaire for the assessment of Alexithymia and it consists of 20 items, divided into three subscales, such as: difficulties in identifying feelings (TAS 1), difficulties in describing feelings (TAS 2) and externally oriented thinking (TAS 3). A 5-point Likert scale is used, and ratings vary from strongly disagree to strongly agree (Herbert et al., 2011). Participants with a TAS total score ≥60 are considered as alexithymic in a clinical manner (Taylor et al., 1991).

#### RHI Experiment

The RHI experiment assesses the sense of body-ownership, or in other words the multisensory integration of tactile, visual, and proprioceptive information (Grynberg and Pollatos, 2015). The degree of proprioceptive drift, the subjective reports of the illusion and the participant’s hand temperature were taken into account in the current study.

For this reason, each adolescent was seated comfortably in front of a table, facing a two-chambered box [size: 36.5 cm × 19 cm × 29 cm (width × height × depth)] with open sides, being unaware of the hypothesis of the study. Moreover, the participant placed his/her left hand into the left chamber and a realistic rubber hand, in means of skin color, texture, and shape, was placed in the right chamber of the box. The participant’s posture was in this way, that he/she could observe the rubber hand throughout the experiment and participant’s middle finger was lightly fixed to the box with a hook and loop fastener. A hairdressers’ cloth was also used, in order to ensure that the participant’s sight was concentrated on the rubber hand. Before each block, participants had to verbally specify the felt location of their left index finger on a ruler placed on the cover of the box, something which is also known as pre-induction proprioceptive location judgment (Tsakiris et al., 2011). Skin temperature was measured at the knuckle of participants’ left index finger with an Infrared Thermometer (Maplin, UK). Furthermore, the synchronous stroking (120 s) of the index finger of the rubber hand and the participant’s hand took place, followed by the assessment of the post-induction temperature and participant’s post-induction proprioceptive location judgment according to a ruler. Meanwhile, the participant was allowed to remove his/her hand from the chamber, and had to fill in the adapted version of the eight items from the RHI Questionnaire for the synchronous condition (Longo et al., 2008). In specific, five items were referred to *ownership* (items 1 to 5; e.g.: “It seemed like the rubber hand was part of my body”) and three items to *location* (items 6 to 8; e.g.: “It seemed like the rubber hand was in the location where my hand was”) (Longo et al., 2008). Moreover, the second block was consisted of the same measurement, but in this case the stimulation of the real and fake hand was made in an asynchronous way and the participant had to complete the same questionnaires, but for the asynchronous condition. The proprioceptive drift for the synchronous and asynchronous induction was calculated in the end of each measurement as the difference between the perceived location of the left index finger before and after the stroking (Tsakiris et al., 2011).

### Statistical Analyses

All statistical analyses were conducted using Statistical Package for Social Sciences (SPSS, version 21). A normality test was performed in order to determine if there was a normal distribution using the Kolmogorov-Smirnov Test. Because of the normal data distribution, parametric tests were conducted, such as Pearson *r* correlations, in order to assess whether there was an interaction between proprioceptive drift and alexithymia. A *p-*value less than 0.05 was considered significant. Continuous variables were summarized as mean and standard deviation; frequencies were used to analyze nominal and ordinal variables. Continuous variables were compared using the two sample *t*-test and analysis of variance (ANOVA). A repeated-measures ANOVA was performed, in order to determine the effect between conditions. One-sample *t*-tests were run, in order to prove if the RHI paradigm produced illusion among adolescents and to confirm the specificity. Multiple regression analysis was performed using the stepwise method, in order to predict the strength of association between all variables of interest. We did not perform a factor analysis due to the small sample size (Brace et al., 2016).

## Results

### Sample Characteristics

Our sample (*N* = 54) was consisted of 28 girls; the average age was 14 years (*SD* = 1.57) and the mean BMI was 19.72 (*SD* = 2.79). **Table 1** demonstrates sample characteristics and questionnaire data concerning alexithymia, emotional distress, and emotional and conduct problems between the younger-older age group, but also among boys and girls.

**Table 1:** Sample characteristics and comparison of means regarding age and gender in all variables of interest.

	*N* = 54	Age			Gender		
			
		Younger	Older	Younger × Older	Girls	Boys	Girls × Boys
	M *(SD)*	M *(SD)*	M *(SD)*	*t*	M *(SD)*	M *(SD)*	*t*
TAS 1	16.20 (4.11)	15.90 (4.25)	16.54 (3.97)	0.57	17.14 (3.55)	15.15 (4.46)	1.82^1^
TAS 2	11.24 (3.50)	11.30 (3.68)	11.17 (3.30)	0.14	11.50 (3.73)	10.96 (3.27)	0.56
TAS 3	22.61 (3.60)	22.30 (3.79)	23.00 (3.36)	0.71	22.25 (2.87)	23.00 (4.26)	0.76
TAS	50.05 (7.40)	49.50 (8.16)	50.71 (6.41)	0.60	50.89 (7.04)	49.11 (7.78)	0.88
EmoDis	0.70 (0.40)	0.63 (0.40)	0.75 (0.40)	1.01	0.75 (0.40)	0.59 (0.39)	1.73^1^
SDQ	11.28 (5.41)	10.00 (4.21)	12.88 (6.35)	1.99^∗^	11‘.68 (5,21)	10.85 (5.70)	0.56


*All values are mean (*SD*) unless stated otherwise; *SD* = Standard Deviation; *t* = independent *t*-test; TAS 1: difficulty identifying feelings; TAS 2: difficulty describing feelings; TAS 3: externally oriented thinking; TAS: total score of the TAS-20; EmoDis: emotional distress measured by the SPS-J; emotional and conduct problems assessed by the SDQ; *p* < 0.05 is considered significant; ^1^trend toward significance. Younger × Older: Interaction between younger (12-14) and older (15-17) age group; Boys × Girls: Interaction between boys and girls. ^∗^*p* < 0.05.*

### Interrelations between Alexithymia, Emotional Distress, and Emotional and Conduct Problems

Emotional and conduct problems together with emotional distress significantly predicted TAS 1, *F*(2,51) = 12.01, *p* < 0.00, adjusted *R*^2^ = 0.32; TAS 2, *F*(2,51) = 6.74, *p* < 0.00, adjusted *R*^2^ = 0.21; TAS, *F*(4,49) = 7.56, *p* < 0.00, adjusted *R*^2^ = 0.38; but not TAS 3, *F*(2,51) = 0.63, *p* > 0.05. Results of the multiple regression analysis are presented in **Table 2**.

**Table 2:** Multiple linear regression analysis predicting alexithymia (subscales and total score) related to emotional distress and emotional and conduct problems.

Outcome	Predictor	*B*	*SE*	β	95 % CI
TAS 1	SDQ	0.32	0.09	0.32^∗∗^	[0.05, 0.42]
	EmoDis	0.38	1.24	0.38^∗∗^	[1.38, 6.36]
TAS 2	SDQ	0.13	0.08	0.20	[-0.04, 0.30]
	EmoDis	3.09	1.14	0.36^∗∗^	[0.80, 5.38]
TAS 3	SDQ	0.10	0.10	0.31	[-0.09, 0.29]
	EmoDis	0.16	1.30	0.90	[-2.46, 2.78]
TAS	SDQ	0.42	0.16	0.31^∗∗^	[0.09, 0.75]
	EmoDis	6.81	2.21	0.37^∗∗∗^	[2.36, 11.26]


*TAS 1: difficulty identifying feelings; TAS 2: difficulty describing feelings; TAS 3: externally oriented thinking; TAS: total score of the TAS-20; EmoDis, emotional distress measured by the SPS-J; emotional and conduct problems assessed by the SDQ; ^∗∗∗^*p* < 0.001; ^∗∗^*p* < 0.01; ^∗^*p* < 0.05.*

### Rubber Hand Paradigm

#### Subjective Ratings

**FIGURE 1:**
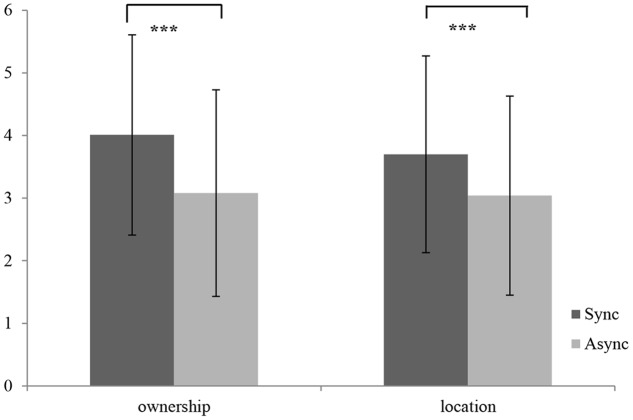
**Subjective ratings of ownership and location after synchronous and asynchronous stimulation as assessed by the RHI Questionnaire; ^∗^*p* < 0.05, ^∗∗^*p* < 0.01, ^∗∗∗^*p* < 0.001**.

For the statistical analysis of the subjective reports, we recoded the answers (from -3 to 3) into new variables (from 1 to 7), where higher values indicate a higher subjective illusion. Moreover, we conducted a one-way repeated-measures ANOVA, setting condition (synchronous vs. asynchronous) as within-subject factor. The results show that the synchronous condition differentiated significantly from the asynchronous one [*F*(1,53) = 19.04, *p* = 0.00, η^2^ = 0.25] indicating a stronger illusion in the synchronous condition. Both mean scores of the subjective ratings regarding *ownership* [synchronous: (*M* = 4.01, *SD* = 1.60) vs. asynchronous: (*M* = 3.08, *SD* = 1.65)] as well as regarding *location* [synchronous: (*M* = 3.70, *SD* = 1.57) vs. asynchronous: (*M* = 3.04, *SD* = 1.59)] were significantly higher after synchronous stimulation (see **Figure 1**) and both conditions did differ significantly [location: *F*(1,53) = 11.23, *p* = 0.00, η^2^ = 0.17] [ownership: *F*(1,53) = 20.46, *p* = 0.00, η^2^ = 0.27].

Boys (*M* = 3.90, *SD* = 1.53) and girls (*M* = 4.11, *SD* = 1.66) did not differentiate in *ownership* and in *location* (boys: *M* = 3.78, *SD* = 1.60; girls: *M* = 3.56, *SD* = 1.55). Additionally, age did not seem to influence the subjective assessment of the body illusion [*ownership*: young (*M* = 4.30, *SD* = 1.58), old (*M* = 3.65, *SD* = 1.56); *location*: young (*M* = 3.95, *SD* = 1.58), old (*M* = 3.30, *SD* = 1.50)].

### Proprioceptive and Temperature Drift

First, proprioceptive drift was calculated by subtracting the position of the finger reported by the participant before the stimulation from the position of the finger reported by the participant after the stimulation. Higher values indicate a higher drift toward the rubber hand. The temperature drift is measured by subtracting the temperature of the finger before the stimulation from its temperature after the stimulation. Lower values indicate a higher drift toward the rubber hand (Grynberg and Pollatos, 2015). After conducting a one-way repeated-measures ANOVA, in order to confirm the specificity of the illusion, we found that proprioceptive drift in the synchronous condition (*M* = 1.14 cm, *SD* = 4.56) did significantly differ in comparison to the asynchronous condition [(*M* = 0.02 cm, *SD* = 2.74); *F*(1,53) = 4.82, *p* = 0.03, η^2^ = 0.08], indicating a significantly stronger body illusion after synchronous stimulation. Thus, we solely focus our further analyses on the synchronous condition. There was an effect of condition on the temperature drift, after conducting a paired-sample *t*-test [*t*(53) = 2.61, *p* = 0.01], suggesting that these dimensions were differentially present. Importantly, a higher drift in the synchronous condition was associated with a higher temperature drop (*r* = -0.30, *p* = 0.02).

Neither gender [(boys: *M* = 0.07, *SD* = 3.66), (girls: *M* = 2.12, *SD* = 5.13); *t*(52) = 1.67, *p* = 0.10], nor age [(younger: *M* = 1.76, *SD* = 5.06), (older: *M* = 0.35, *SD* = 3.80); *t*(52) = 1.13, *p* = 0.26] influenced the degree of proprioceptive drift in the synchronous condition. Furthermore, **Table 3** demonstrates the correlations between proprioceptive drift, temperature, subjective reports and questionnaire data.

**Table 3:** Pearson *r* correlation matrix for the RHI (subjective and objective reports) and questionnaire data

	Drift_S	Temperature_S	Ownership_S	Location_S	EmoDis	SDQ
Drift_S	1.00					
Temp_S	-0.40^∗∗^	1.00				
Ownership_S	0.35^∗∗^	-0.19	1.00			
Location_S	0.27^∗^	-0.01	0.69^∗∗^	1.00		
EmoDis	0.10	-0.02	0.14	-0.01	1.00	
SDQ	0.26^∗^	-0.45^∗∗^	0.21	0.18	0.32^∗^	1.00


*Drift_S: proprioceptive drift in the synchronous condition; Temp_S: Temperature drift in the synchronous condition; Ownership_S: subjective report of body ownership in the synchronous condition; Location_S: subjective report of location in the synchronous condition; EmoDis: emotional distress measured by the SPS-J; emotional and conduct problems assessed by the SDQ; ^∗∗∗^*p* < 0.001; ^∗∗^*p* < 0.01; ^∗^*p* < 0.05.*

### Alexithymia and RHI

**FIGURE 2:**
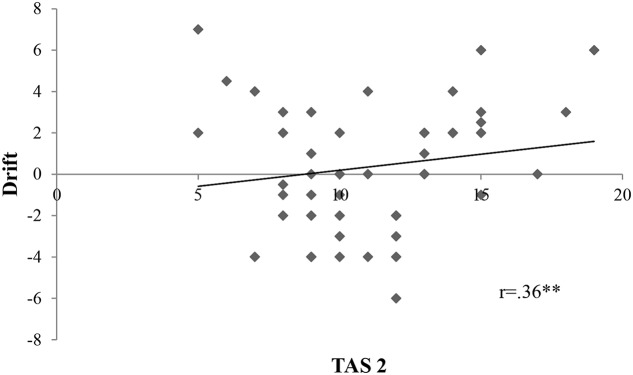
**Scatterplot depicting the interrelation between TAS 2 and proprioceptive drift; Drift, proprioceptive drift in the synchronous condition; TAS 2, “difficulties in describing feelings”.** There is a statistically significant positive correlation between drift and TAS 2 with a *p*-value of 0.01; ^∗^*p* < 0.05, ^∗∗^*p* < 0.01, ^∗∗∗^*p* < 0.001; *r* = Pearson *r* correlation coefficient.

**FIGURE 3:**
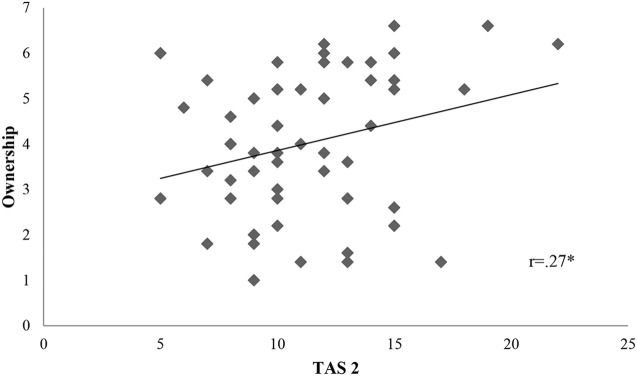
**Scatterplot portraying the interaction between TAS 2 and Ownership.** Ownership, subjective rating of *ownership* in the synchronous condition. TAS 2, “difficulties in describing feelings”. There is a statistically significant positive correlation between ownership and TAS 2 with a *p*-value of 0.05; ^∗^*p* < 0.05, ^∗∗^*p* < 0.01, ^∗∗∗^*p* < 0.001; *r* = Pearson *r* correlation coefficient.

We calculated firstly Pearson *r* correlations between all variables of interest. Proprioceptive drift was associated to TAS 2 subscale (*r* = 0.36, *p* = 0.01) (**Figure 2**). *Ownership* was connected only to TAS 2 (*r* = 0.27, *p* = 0.05) (**Figure 3**). On the contrary, *location* was not related to the subscales of alexithymia. Finally, temperature drift was not connected to the facets of alexithymia [TAS 1: *r* = -0.06, *p* = 0.69 ; TAS 2: *r* = -0.16, *p* = 0.24; TAS 3: *r* = -0.07, *p* = 0.59].

We further on conducted a multiple regression using the stepwise method, setting as outcome the different RHI measures (objective and subjective) and as predictors the alexithymia subscales, emotional distress, emotional and conduct problems. **Table 4** shows the results of this analysis. As expected, we found significant associations between ownership and TAS 2 [*F*(1,52) = 4.06, *p* = 0.05, *R*^2^ = 0.07] and between drift and TAS 2 [*F*(1,52) = 7.64, *p* = 0.01, *R*^2^ = 0.13].

**Table 4:** Regression analysis predicting TAS 2 on the basis of RHI subjective and objective measures.

Outcome	Predictor	*B*	*SE*	*t*	95% CIs
Model 1					
Ownership_S	TAS 2	0.12	0.06	2.02^∗^	[0.00, 0.24]
Model 1					
Drift_S	TAS 2	0.47	0.017	2.76^∗∗^	[0.13, 0.81]


*Drift_S: proprioceptive drift in the synchronous condition; Ownership_S: subjective report of body ownership in the synchronous condition; TAS 2: difficulty describing feelings; ^∗∗∗^*p* < 0.001. ^∗∗^*p* < 0.01; ^∗^*p* < 0.05.*

## Discussion

Scope of this study was to investigate the malleability of body ownership using the RHI experiment and its interrelation to alexithymia in adolescence. First, findings of this study confirmed the feasibility of the RHI paradigm in adolescents regarding objective and subjective indices of body illusion and further interrelations between these measures were found. The main result was that one emotional facet of alexithymia, namely difficulties in describing feelings (TAS 2; DDF), was associated with a stronger malleability of body-ownership as reflected both in objective markers (proprioceptive drift) as well as in subjective reports (items on body-ownership).

More specifically, the subjective ratings concerning body illusion indicated that the degree of illusion regarding ownership and location was greater in the synchronous condition than in the asynchronous, something that is consistent with previous literature among adults (Tsakiris et al., 2011; Grynberg and Pollatos, 2015; Riemer et al., 2015). Likewise, the above was confirmed at a behavioral level, in which participants perceived their hand closer to the rubber hand after the synchronous induction in contrast to the asynchronous, and the temperature drop (autonomic measure) after synchronous visuo-tactile stimulation was connected to a larger proprioceptive drift. This is in agreement with previous research (Tsakiris et al., 2011) showing that both objective measures of the body illusion are interconnected. Hence, that could lead us to the conclusion that the integration of visual and tactile information in order to influence proprioception is feasible among adolescents, as measured by the RHI, and that the present results could replicate the original illusion, not only at a behavioral level, but also at a subjective and autonomic level, as has been previously shown in adults (Botvinick and Cohen, 1998; Tsakiris et al., 2011; Grynberg and Pollatos, 2015) and in children (Cascio et al., 2012).

Surprisingly, gender and age did not influence subjective and objective ratings in the RHI. Body dissatisfaction is known to be more pronounced in females, where girls have a tendency to report more negative body image than boys, something which is consistent with the thin ideal of girls in the European countries (Holubcikova et al., 2015). We did not observe a greater malleability of body representation in girls as assessed by the RHI. One important point to consider is that our sample consisted of normal-weighted adolescents with presumably no or rather low amount of eating problems, suggesting that alternations in body image might be rather small. As an unhealthy body image is often associated with sedentary lifestyle, obesity and eating disorders (Voelker et al., 2015), additional sample of adolescents with a larger range concerning body weight might change the picture.

Karukivi et al. (2014a,b) suggest that alexithymia is a stable personality trait among adolescents, which can be linked up to lack of social support from friends and to an intrusive and overprotective parenting. From another point of view, alexithymia might also be perceived as a developmental characteristic in young children and not as a pathological phenomenon (Tolmunen et al., 2010). Interestingly, our results revealed that emotional and conduct problems as assessed by the SDQ predicted DIF, whereas emotional distress predicted the emotional aspects of alexithymia (DIF/DDF subscales), highlighting the close link between a possible psychopathology and alexithymic behavior connected to emotion awareness. This finding could highlight problems in affect regulation associated with alexithymia as demonstrated in adults (Taylor et al., 1999; Taylor and Bagby, 2004; Foran and O’Leary, 2013; Panayiotou et al., 2015). Furthermore, the idea that a disturbed body ownership could be related to emotional or conduct problems, was also supported by the fact that SDQ mean scores and proprioceptive drift were associated. Due to the lack of studies in this field, further research is required to establish a conclusion and to determine the specificity of the psychopathologic symptoms in this age.

Main finding of this study in adolescents was that DDF predicted proprioceptive drift and the subjective report of ownership in the RHI. DDF belong to the emotional part of alexithymia and describe a crucial ability for social communication, allowing fine-tuned feedback in emotional situations. There is further evidence that alexithymic individuals face difficulties in differentiating affective symptoms from somatic, which are related to interoceptive experiences (Byrne and Ditto, 2005; Panayiotou et al., 2015). Furthermore, Panayiotou et al. (2015) suggested that alexithymia is linked to the characteristic of experiential avoidance (EA), which is one’s effort to avoid experiencing unpleasant emotions, memories, or aversive bodily sensations. On the other hand, previous evidence has also revealed a connection between deficits in the perception, processing, and interpretation of verbal and nonverbal emotional stimuli in alexithymia (Roedema and Simons, 1999; Lane et al., 2000; Stone and Nielson, 2001; Berthoz et al., 2002; Kano et al., 2003; Luminet et al., 2006; Pollatos et al., 2008; Herbert et al., 2011). In line with these ideas, we suggest that in adolescence, a vicious circle might occur, in which affect and integration of different exteroceptive/proprioceptive bodily signals become more and more entangled. Whether alexithymia leads to an attenuated body ownership among adolescents, or an attenuated body ownership triggers alexithymia, remains yet unclear, as correlational analyses do not allow elucidating the causal chain of our observation.

Nevertheless, possible limitations of this study could be the absence of data regarding personality traits, like introversion or extraversion, which could provide more individual characteristics of the participants. Accordingly, in this study, we used the TAS 20 in order to assess alexithymia; further studies in this field could be conducted on the basis of experimentally assessing alexithymia. Due to the small sample size, exploratory factor analysis was not possible for the investigation of the underlying structure in the pattern of correlations between RHI alexithymia, something that could also be seen as a shortcoming. Therefore future analyses should include a larger sample and could be conducted in a longitudinal fashion during childhood or adolescence.

To sum up, findings of this study suggest that the measurement of multi-sensory integration regarding body ownership among adolescents, arising from vision and touch can be behaviorally and physiologically estimated. We conclude that our results illustrate for the first time an intriguing link between alexithymic traits and malleability of bodily representations in adolescence, assessed by the RHI. As this is to our knowledge the first study investigating these variables among adolescents, more research on this topic needs to be undertaken to further elucidate the obtained associations and to further observe more closely the developmental characteristics that underlie the interplay between the sense of body-ownership and emotion awareness.

## Author Contributions

EG: study conception, study design, data collection, data analysis, preparation of the MS. SM: data collection, MS editing, MS proof reading. OP: study design, data analysis, MS editing, MS proof reading.

## Conflict of Interest Statement

The authors declare that the research was conducted in the absence of any commercial or financial relationships that could be construed as a potential conflict of interest.
